# New adjustable modular hemipelvic prosthesis replacement with 3D-print osteotomy guide plate used in periacetabular malignant tumors: a retrospective case series

**DOI:** 10.1186/s13018-022-03150-0

**Published:** 2022-05-12

**Authors:** Jun Li, Zicheng Liu, Dan Peng, Xia Chen, Chao Yu, Yi Shen

**Affiliations:** Department of Orthopedics, The Second Xiangya Hospital of Central South University, Changsha, 410000 Hunan China

**Keywords:** Periacetabular malignancies, Hemipelvic replacement, 3D-print, Functional recovery, Dislocation

## Abstract

**Background:**

Periacetabular malignant tumor seriously endangers the life and health of patients. Hemipelvic replacement provides a good method for patients who want complete resection of the tumor while retaining or restoring the function of the affected limb.

**Objective:**

To investigate the performance and clinical application of the new adjustable modular hemipelvic prosthesis and to compare the effects of three kinds of hemipelvic prosthesis.

**Methods:**

In this study, 23 patients, with an average age of 44.6 years (21–75 years), were collected, who received hemipelvic replacement with new adjustable, modular, and screw-rod system hemipelvic prosthesis. Preoperative preparation was conducted on them, and operative complications were recorded. Postoperative functional follow-up was performed regularly.

**Results:**

The average operation time was 319 min (170–480 min), and the average blood loss was 2813 ml (1000 mL-8000 ml). The incidence of complications was 47.8%, and type A (wound-related complications) had the highest incidence (34.8%). Postoperative dislocation occurred in 3 cases (13.0%), and no dislocation occurred in the new adjustable modular hemipelvic prosthesis group. The average MSTS score of the patients was 18.6 (10–23), and the average Harris score was 73.7 (53–87).

**Conclusions:**

The new adjustable modular hemipelvic prosthesis has the feasibility of reconstruction and good functional outcome, making it ideal for periacetabular tumors. Furthermore, preoperative tumor-feeding artery embolization and abdominal aortic balloon implantation may be an effective choice to reduce intraoperative blood loss and facilitate the operation of tumor resection.

## Background

The accurate and appropriate treatment of pelvic tumors not only affects the life span of patients but also profoundly affects the quality of life of patients [1, 2]. The pelvis is a rare neoplastic site, and the most common pathological type is metastatic tumors [3]. Due to the deep and hidden location, the large pelvic defects after mass resection lead to the lack of intraoperative bony anatomical markers, which brings operation with more difficulties, more bleeding, longer operation time, and poorer prognosis than bone tumors in other parts [4, 5]. Nowadays, surgery is still the best way to treat pelvic tumors, and hemipelvic replacement provides a superb method [6, 7]. The hemipelvic prosthesis used in surgery mainly includes screw-rod hemipelvic prosthesis, modular hemipelvic prosthesis, 3D printing hemipelvic prosthesis, custom-made personalized hemipelvic prosthesis, and so on [4, 8–12]. The advantages of hemipelvic replacement are that not only the tumor can be completely removed, but also the hip can be reconstructed to retain or restore lower limb function [13]. Therefore, it can improve the survival rate and quality of life of patients. In addition, preoperative imaging technology, navigation technology, 3D printing technology, new orthopedic implants, arterial infusion chemotherapy, molecular targeted therapy, and other technologies have gradually improved the efficacy of pelvic therapy [4, 12, 14–16].

In this study, our team developed and applied a new adjustable modular hemipelvic prosthesis to treat periacetabular malignancies combined with a 3D-print osteotomy guide plate. This prosthesis is designed to adapt to different osteotomy ranges. Therefore, it can better restore the center of rotation of the pelvis to reduce the rate of dislocation and restore function.

## Methods

### Patient information

This retrospective study was approved by the Medical Ethics Committee of The Second Xiangya Hospital. Meanwhile, all the patients signed written informed consents. Inclusion criteria: (1) tumors involved in region II of the pelvis (acetabulum); (2) malignant tumors included primary and metastatic bone tumors. Exclusion criteria: (1) the patient's cardiopulmonary function cannot tolerate such a complex operation; (2) the tumors involve the iliac vessels or sciatic nerve or the surrounding important organs, making it difficult to obtain complete resection; 3) the patients had extensive metastases that were difficult to resect completely and had an estimated life span of less than 1 year. In this study, a total of 23 patients, including 15 males and 8 females, with an average age of 44.6 years (21–75 years) were collected. All the patients received hemipelvic replacement from January 2012 to January 2020. These patients were admitted to the hospital with chief complaints of “pain, limited mobility, and mass.” The pathological types were confirmed via preoperative biopsy including 9 chondrosarcomas, 4 GCCTs (giant cell tumor of bone), 3 angiosarcomas, 2 mesenchymal malignancies, 1 fibrosarcoma, 1 osteosarcoma, 1 synovial sarcoma, 1 invasive chondroblastoma, and 1 metastatic lung adenocarcinoma. All patients received three kinds of hemipelvic replacement surgeries, respectively, including 5 patients with new adjustable modular hemipelvic replacement, 12 patients with modular hemipelvic replacement, and 6 patients with screw-rod system hemipelvic replacement. The characteristics and outcomes of 23 patients with malignant pelvic tumors are given in Table 1.

**Table 1:** Characteristics and outcomes of 23 patients with malignant pelvic tumors

Case	Gender	Age	Reconstruction methods	Surgical margin	Pathological diagnosis	Stage	Resection type	Intervention operation	Blood loss (ml)	Complications	LLD(cm)	Follow-up (months)	MSTS score	Harris score	Recurrence metastasis	Patient status
1	Male	54	NAHP	Wide	Angiosarcoma	IIB	I + II	YES	1200	Infection	1	21	20	80	Recurrence	DOD
2	Male	65	NAHP	Wide	Metastatic lung adenocarcinoma	III	II + III	YES	1400		1	26	17	66		AWD
3	Male	46	NAHP	Marginal	Angiosarcoma	IIB	I + II	YES	2500	Pulmonary	1.5	27	19	77		NED
4	Female	53	NAHP	Wide	Chondrosarcoma	IIB	I + II	YES	1200	Infection	1	23	23	83		NED
5	Female	56	NAHP	Wide	Mesenchymal malignancy	IIB	I + II	YES	1000		1	21	22	85		NED
6	Female	38	MHP	Marginal	mesenchymal malignancy	IIB	I + II + III	NO	2000	Cutaneous necrosis	1.5	20	15	57	Lung Metastasis	DOD
7	Male	33	MHP	Wide	MGCT	IIB	II	NO	1500		1	58	23	87		NED
8	Female	51	MHP	Wide	Chondrosarcoma	IIB	II + III	NO	2600		1	19	19	76	Recurrence	DOD
9	Male	35	MHP	Marginal	MGCT	IIB	II + III	NO	3200		2	46	14	53		NED
10	Male	28	MHP	Wide	Synovial sarcoma	IIB	I + II + III	NO	2100	Dislocation	2.5	52	22	84		NED
11	Male	34	MHP	Wide	Invasive chondroblastoma	IIB	II + III	NO	5000	Wound dehiscence	2	45	20	77		NED
12	Female	45	MHP	Wide	Chondrosarcoma	IIB	II + III	NO	4800	Wound dehiscence	1	26	18	73		NED
13	Female	27	MHP	Wide	Chondrosarcoma	IIB	II + III	NO	2700		1.5	28	18	76		NED
14	Female	50	MHP	Wide	Chondrosarcoma	IIB	II + III	NO	2500		1	19	21	83		NED
15	Male	45	MHP	Wide	MGCT	IIB	I + II + III	NO	8000	Infection	NA	36	10	56		NED
										Tension blisters						
										HA						
16	Male	37	MHP	Wide	fibrosarcoma	IIB	I + II	NO	5000		1	37	23	85		NED
17	Male	45	MHP	Wide	MGCT	IIB	II + III	YES	2300		1.5	47	20	78		NED
18	Male	48	S-RHP	Marginal	Angiosarcoma	IIB	I + II	NO	3200	Infection	1	26	18	75	Recurrence	DOD
19	Male	57	S-RHP	Wide	Chondrosarcoma	IIB	I + II	NO	3700	Wound dehiscence	1.5	25	14	56		NED
20	Male	75	S-RHP	Marginal	chondrosarcoma	IIB	I + II	YES	3000	Infection	2	18	17	72	Recurrence	DOD
										Dislocation					Lung Metastasis	
										Urinary retention						
										Thrombosis						
21	Male	61	S-RHP	Wide	chondrosarcoma	IIB	I + II	NO	2600	Dislocation	1	20	15	61	Recurrence	DOD
22	Female	21	S-RHP	Wide	chondrosarcoma	IB	I + II	NO	1200		1.5	38	19	80		NED
23	Male	21	S-RHP	Wide	osteosarcoma	IIB	I + II	NO	2000		1.5	21	20	76	Recurrence	DOD
															Lung Metastasis	

NAHP: new adjustable modular hemipelvic prosthesis, MHP: modular hemipelvic prosthesis, S-RHP: screw-rod hemipelvic prosthesis, MGCT: malignant giant cell tumor,

HA: hip amputation, NED: no evidence of disease, DOD: died of disease, AWD: alive with disease, NA: not available, MSTS: Musculoskeletal Tumor Society

### Preoperative preparation and operation

**Fig. 1:**
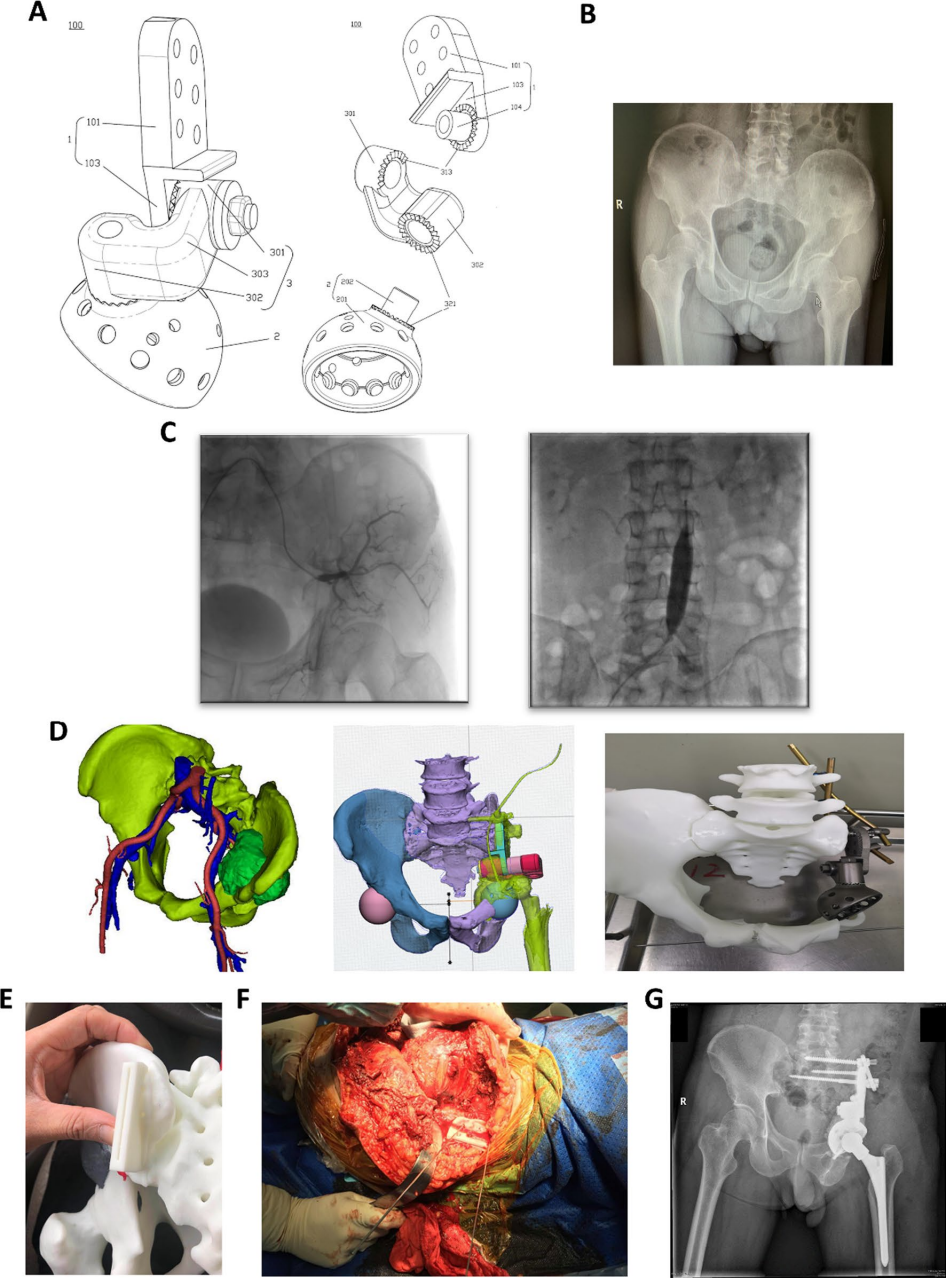
A typical case of the new adjustable modular hemipelvic replacement. **A** Design schematic diagram of the new adjustable modular hemipelvic prosthesis. **B** Preoperative radiographs of a 54-year-old man showing angiosarcoma of the left pelvis involving acetabulum and ilium. **C** Preoperative abdominal aortic balloon placement and embolization of tumor-feeding artery and internal iliac artery. **D** Preoperative 3D printing model of pelvic tumor and simulated surgery. **E, F** Precise and complete resection of the tumor guided by a custom osteotomy guide plate. **G** Postoperative radiographs of a 54-year-old man showing malignant angiosarcoma of the left pelvis involving acetabulum and ilium

Pelvic X-ray, lung CT, pelvic enhanced CT, pelvic enhanced MRI, and whole-body bone scan were performed before surgery. The pelvic tumor was zoned according to the Enneking system to guide the choice of the surgical incision and surgical scope. There were 11 cases in regions I + II, 8 cases in regions II + III, 3 cases in regions I + II + III, and 1 case in region II. For the modular and new adjustable modular hemipelvic prosthesis, it is also necessary to conduct a 3D reconstruction of the pelvic CT. Furthermore, the tumor model is 3D-printed preoperatively to determine the osteotomy plane and customize the osteotomy guide plate according to the individual situation (Fig. 1D, E, F). For the new adjustable modular hemipelvic prosthesis, appropriate specifications of the steering column (structure 3) ranging from 0 to 50 mm were selected according to the condition of the residual pelvis to individualize the rotation center of the acetabulum (Fig. 1A). Before surgery, intestinal preparation was performed according to the requirements of colon surgery, and a double-J tube was placed by the experienced urology surgeon. Some patients underwent abdominal aortic balloon placement and embolization of tumor-feeding artery and internal iliac artery to reduce surgical bleeding (Fig. 1C).

**Fig. 2:**
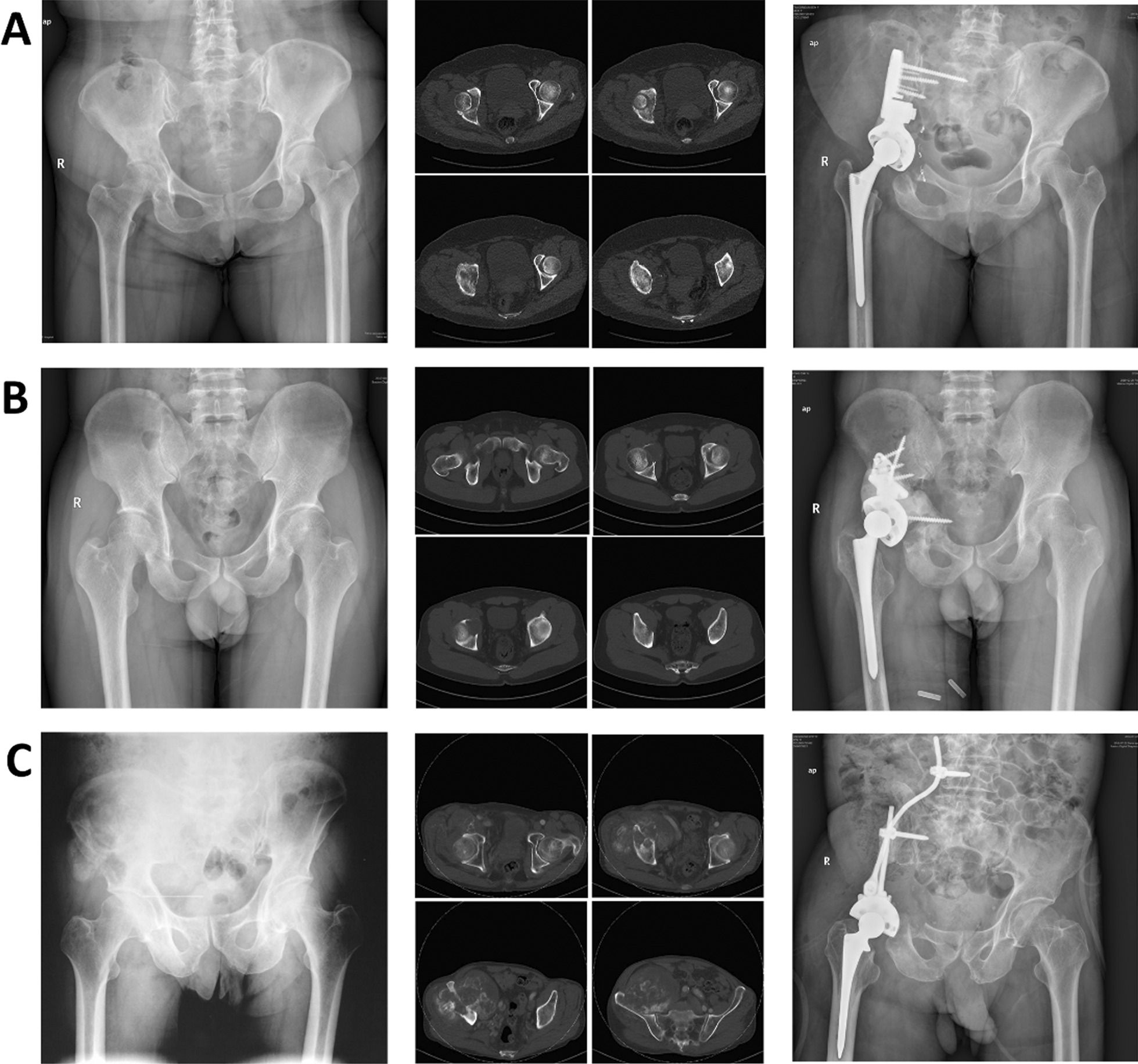
Typical cases of three kinds of hemipelvic prostheses. **A** Radiographs of a 53-year-old woman showing chondrosarcoma of the right pelvis involving acetabulum and ilium (Case 4 in Table1). **B** Radiographs of a 33-year-old man showing a malignant giant cell tumor of the right pelvis involving the acetabulum (Case 7 in Table1). **C** Radiographs of a 75-year-old man showing dedifferentiated chondrosarcoma of the right pelvis involving acetabulum and ilium (Case 20 in Table1)

The patient was placed in the healthy lateral decubitus position and can switch between the lateral supine and lateral prone position. A combined Smith-Petersen and ilioinguinal approach was used. The femoral vessels, femoral nerve, and sciatic nerve were dissociated and protected. The pelvic tumor was completely removed according to the preoperative osteotomy plane. The osteotomy of the NAHP group was completed under the guidance of a 3D-printed osteotomy guide plate. A femoral neck osteotomy was performed to remove the femoral head. An appropriate prosthesis was selected according to the residual pelvic bone structure so that the acetabular cup could be reconstructed in the original acetabulum as much as possible. If necessary, osteotomy was performed again to adjust the position of the prosthesis. Multiple screws were used to fix the prosthesis to the residual ilium, sacrum, pubis, ischium, and lumbar vertebrae, depending on the situation. The prosthesis and adjacent bone were strengthened with bone cement if necessary. A cement or biological femoral prosthesis was then installed, and the hip was reduced. 1 ~ 2 wound drainage tubes were placed, and the wound was sutured according to layers. The typical cases of three kinds of hemipelvic replacements are shown in Figs. 1 and 2.

### Postoperative recovery and follow-up

After surgery, the patients were instructed to wear neutral anti-rotation shoes and strengthen the lower limb muscles by ankle pump exercise and quadriceps contraction. About 4–8 weeks later, weight-bearing exercises were performed as the case may be. The patients with osteosarcoma received standard postoperative chemotherapy. All the patients were followed up every 3 months. Harris score and MSTS score were performed on patients at 1-year follow-up. Postoperative complications were evaluated according to the method described by Zeifang [17]. Zeifang classified the complications into five categories: Type A (wound-related complications), Type B (implant failure), Type C (systemic complications), Type D (others), and Type E (local tumor recurrence).

## Result

The average operation time was 319 min (170–480 min), and the average blood loss was 2813 ml (1000–8000 ml). Seven patients underwent abdominal aortic balloon implantation and embolization of tumor-feeding artery and internal iliac artery, and the average blood loss of these patients was 1800 ml (1000–3000 ml).

The postoperative complications are given in Table 2.

- **Type A:** The incidence of type A was the highest, accounting for 34.8% (8/23). Among them, there were 4 cases of wound infection, 3 cases of wound dehiscence, and 1 case of skin necrosis. Three patients with wound dehiscence received healing after dressing change. Four patients received healing after debridement and negative pressure wound therapy. Another patient developed a large area of tension blisters which progressed to periprosthetic infection. Two months later, hip disarticulation was conducted.
- **Type B**: There were 3 cases of dislocation, including 1 case of modular hemipelvic prosthesis and 2 cases of screw-rod hemipelvic prosthesis. All the dislocations were caused by improper postoperative position. After dislocation, one patient underwent closed reduction and one patient received open reduction under general anesthesia. Another patient gave up treatment due to recurrence. No complications such as prosthesis fracture and loosening occurred during the follow-up.
- **Type C:** One patient developed a pulmonary infection and was admitted to ICU for 1 week. Because of passive movement and autonomous functional exercise in the early stage, only one 75-year-old patient developed lower limb deep vein thrombosis and urinary retention.
- **Type D:** All the patients and their family members were advised to turn over and pay close attention to skin condition, no bedsore occurred. The average difference in the length of both legs after surgery was 1.4 cm (1–2.5 cm), and over 2 cm is considered unacceptable.
- **Type E:** Four patients (2 chondrosarcomas and 2 angiosarcomas) had local recurrence, and one patient with mesenchymal malignancy had lung metastasis. Two patients (1 osteosarcoma and 1 chondrosarcoma) had both local recurrence and lung metastasis.

**Table 2:** Complications and score of different hemipelvic prostheses in 23 patients

Variables	NAHP	MHP	S-RHP	In all
Complications (person-time)				
Type A	1	4	3	8
Type B	0	1	2	3
Type C	1	0	1	2
Type D	0	1	0	1
Type E	1	2	4	7
Score (point)				
MSTS	20.2	18.6	17.2	18.6
Harris	78.2	73.8	70.0	73.7

**Fig. 3:**
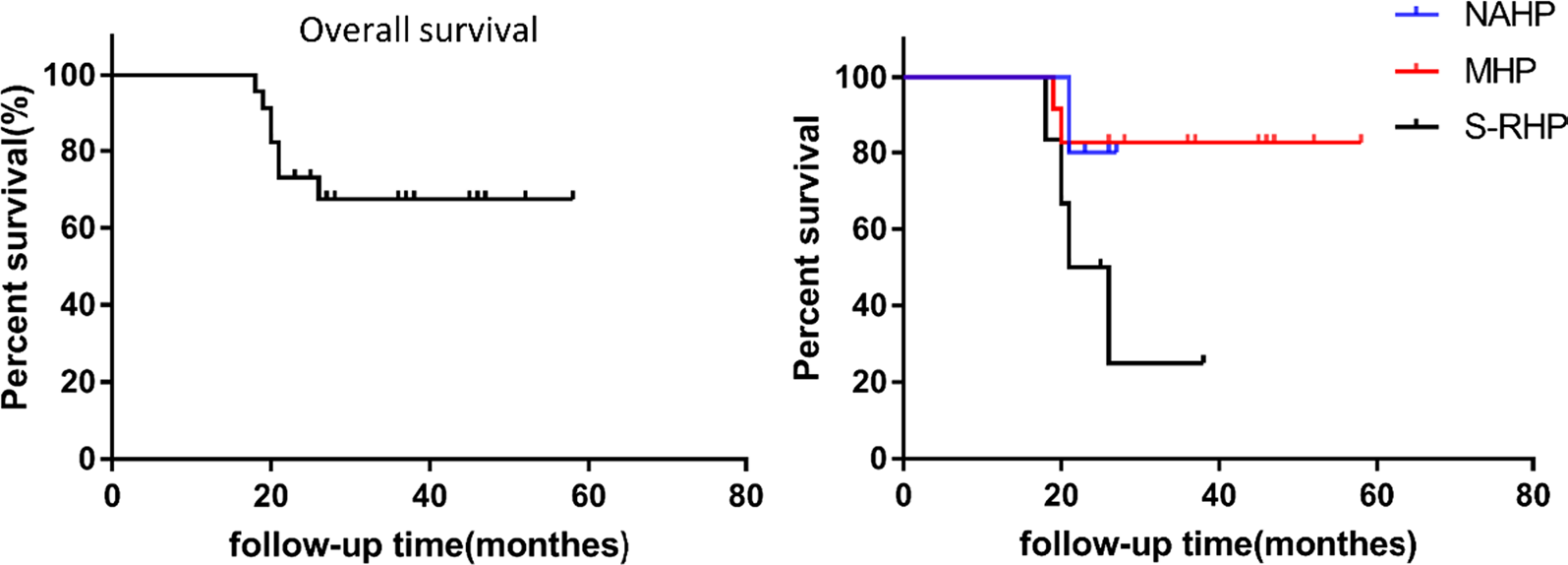
The overall and separate survival rates in 23 patients

The average follow-up time of the 23 patients was 30.4 months (18–58 months). The Harris score (full score of 100) and MSTS 93 score (full score of 30) were recorded at 1-year follow-up [18]. The average MSTS score was 18.6 (10–23), and the average postoperative Harris score was 73.7 (53–87). Postoperative scores of the three surgical methods are given in Table 2. The overall 1-year survival rate was 100%, and the 3-year survival rate was 67.4% (Fig. 3).

## Discussion

Hemipelvic replacement provides a good method for patients who want complete resection of the tumor while retaining or restoring the function of the affected limb [19]. The design trend of the hemipelvic prosthesis will be stable, convenient, biocompatible, and personalized.

Removal of pelvic tumors will result in bone defects. Therefore, a variety of hemipelvic prostheses have been developed for pelvic reconstruction. Due to the different location and size of the pelvic tumor, the degree of the bone defect after tumor resection is different [16]. However, the existing modular pelvic prosthesis cannot meet the good applicability of patients with different degrees of bone defect and cannot well achieve the personalized recovery of the acetabular rotation center. As shown in Fig. 1A, the new adjustable modular hemipelvic prosthesis developed by us may solve the problem (Patent no. CN201921588367.4). Horizontal and vertical displacement of the acetabular cup can be regulated by choosing a suitable steering column. (Both arms of the steering column are available in a range of sizes ranging from 0 to 50 mm.) Furthermore, the anteversion and abduction angles can be restored by adjusting the angle between the steering column (structure 3) and the pelvic seat (structure 1) and the angle between the steering column (structure 3) and the acetabular cup (structure 2). As a result, it can increase the applicability of the pelvic prosthesis and fundamentally reduce the dislocation rate. Two prostheses have one thing in common: For pelvic tumors in the acetabulum (region II) or near the acetabulum, both of the two prostheses work well because the big prosthetic pelvis fixation base can be attached to the remaining ilium or sacrum [20]. Compared with the screw-rod system, the new adjustable modular hemipelvic prosthesis has better physical properties and stability and is not easy to loosen and fracture. For pelvic tumors involving the sacroiliac joint or sacrum, the study of Zhang et al. combined the modular hemipelvic prosthesis with the pedicle screw system to obtain a more stable pelvic structure [21]. Furthermore, the study of Liu et al. showed that the biomechanical properties of the bilateral pedicle system are superior to that of the unilateral pedicle system because the bilateral pedicle screw system could make bilateral displacement and stress transfer more uniform in the reconstruction of the pelvis [22]. However, the study of Wang et al. suggested that additional screw fixation in the first sacral vertebra during hemipelvic replacement for periacetabular tumors involving the sacroiliac joint did not improve the short-term follow-up of patients [23]. Therefore, it is very significant to select a suitable prosthesis and strive for good biomechanical reconstruction.

Metastatic carcinoma is the most common malignant tumor of the pelvis, but various primary tumors often occur [4]. About 3% to 4% of primary bone tumors are located in the pelvis, and the pathological types of pelvic tumors are very complex, with adult chondrosarcoma, pediatric Ewing's sarcoma, and adolescent osteosarcoma being the most common histological subtypes [10, 24]. Chondrosarcoma was the most common tumor in our study, which was consistent with most researches because most patients with metastatic pelvic malignancies rarely chose hemipelvic replacement. Prognosis is different for different tumor types. Therefore, it is also very important to give appropriate adjuvant chemotherapy and neoadjuvant chemotherapy according to different pathological types [15]. In addition, the study of Alfredo Guilherme Haack Couto et al. found that the survival rate of patients with bone tumors was significantly higher than those of soft tissue sarcomas, and they found that almost all patients with soft tissue tumors had more advanced tumor stages (stage III or IV), which may be the reason for the decreased survival rate [25].

In this study, type A (wound-related complications) had the highest incidence, which was in line with many studies [7, 26–29]. The incidence of type A is related to operative time, age, basic diseases such as diabetes, blood loss, the volume of drainage, extubation time after surgery, and preoperative antibiotic prophylaxis regimen [4, 17, 30, 31]. Hemipelvic replacement was one of the orthopedic surgeries with the greatest blood loss. In our research, the average blood loss of the patients with balloon placement and embolization was less than that not. In particular, balloon placement is an effective means of dealing with dangerous hemorrhages. According to the study of Luo and Ratto, preoperative abdominal aortic balloon implantation can reduce intraoperative blood loss, make the surgical field clearer, improve surgical safety, and reduce the length of hospital stay and ICU stay without obvious sequelae [9, 32]. In addition, some studies have shown that appropriate lowering of blood pressure during anesthesia can reduce intraoperative blood loss, while not increasing other complications [33]. Therefore, it is very important to fully evaluate the preoperative tumor condition, prepare blood, lower blood pressure appropriately during anesthesia, place an abdominal aortic balloon, and embolize tumor supplying artery or internal iliac artery, particularly for those with angiogenic or metastatic tumors.

Dislocation is a common and troublesome postoperative complication. In this research, postoperative dislocation occurred in 3 cases, and no dislocation occurred in the NAHP group. Those patients suffered from dislocation due to improper postoperative position. In recent years, our team has adopted and patented this new adjustable modular hemipelvic prosthesis as shown in Fig. 1A (Patent no. CN201921588367.4), which is designed to individualize the rotation center of the acetabulum and fundamentally reduce the dislocation rate of the pelvis. In addition, to reduce the occurrence of dislocation, it is also very important to carefully repair the joint capsule and the soft tissues around the joint, prolong the immobilization time, keep the legs slightly outside, avoid unreasonable activities, and improve the prosthesis such as hinge joints and constraint liners [21, 34–37]. Wang et al.'s study indicated that patients were more likely to dislocate in the first 3 months after surgery, and the risk of dislocation is increased in patients with older age (especially those aged > 60 years), gluteus maximum resection, the center of rotation vertical displacement ≥ 20 mm, and type I + II + III pelvic resection [38].

Limitations: (1) because of the rarity of pelvic tumors, the number of cases is relatively small, and the follow-up is mainly short term and medium term. It is more convincing to increase the number of cases and follow-up time to further verify the performance of the prosthesis and postoperative dislocation. (2) Although all the study subjects were patients with periacetabular tumors, there was heterogeneity in tumor type, tumor size, and patient age. (3) NAHP mainly focuses on personalized acetabular reconstruction. It is difficult for NAHP to treat pelvic tumors involving the sacrum, which require the use of special pelvic implants such as 3D-printing prosthesis. (4) For tumors involving the pubis and ischium, NAHP cannot reconstruct the pubis and ischium to restore the integrity of the pelvic ring.

## Conclusion

In conclusion, the new adjustable modular hemipelvic prosthesis adopted by us has the feasibility of reconstruction and good functional outcome, which will be a good and promising hemipelvic prosthesis solution for patients with periacetabular tumors. Furthermore, preoperative tumor-nourishing artery embolization and abdominal aortic balloon implantation may be an effective choice to reduce intraoperative blood loss and facilitate the operation of tumor resection.

## Data Availability

All the relevant data and materials can be found in the tables of the article.

## Abbreviations

NAHP: New adjustable modular hemipelvic prosthesis
MHP: Modular hemipelvic prosthesis
S-RHP: Screw-rod hemipelvic prosthesis
CHP: Custom-made hemipelvic prosthesis
3D-HP: 3D-printed hemipelvic prosthesis
MGCT: Malignant giant cell tumor
HA: Hip amputation
NED: No evidence of disease
DOD: Died of disease
AWD: Alive with disease
NA: Not available
MSTS: Musculoskeletal tumor society
TESS: Toronto extremity salvage score
